# How to express social presence: a comparison of perceptions of social robots’ cognitive abilities

**DOI:** 10.3389/frobt.2026.1938840

**Published:** 2026-10-01

**Authors:** Thomas Sievers, Nele Russwinkel

**Affiliations:** Institute of Information Systems, University of Luebeck, Lübeck, Germany

**Keywords:** anthropomorphism, care, cognition, large language model, memory, social presence, social robot, vision language model

## Abstract

In light of the problems posed by an aging society and the associated challenges–particularly regarding the care and support of older citizens–questions arise concerning technological solutions. Social robots equipped with appropriate cognitive abilities can provide support in this area by reducing loneliness and social isolation through communication and personalized interaction. To this end, the robot’s perceived social presence is of crucial importance. Social presence (or co-presence) refers to the sense of being with another person and depends on how easily one feels able to access that person’s intelligence, intentions, and sensory impressions. This study examined the potential of robots’ cognitive abilities for social dialogic interactions with humans, such as (i) unrestricted conversational abilities, (ii) the incorporation of visual cues into interactions, and (iii) memories of individual encounters, and investigates the perceived social presence in each case. In addition, we evaluated the degree of anthropomorphism using the Human-Robot Interaction Evaluation Scale. Our results show a trend toward a higher degree of social presence in terms of visual perception, but only one statistically significant difference out of a total of seven questions on this topic. Memories, as well, had only one statistically significant influence, namely, on the anthropomorphic dimension of disturbance. Gender-specific differences were observed with regard to social presence and the anthropomorphic dimensions. However, these differences are most likely attributable to professional background or certain predispositions rather than to gender itself.

## 1 Introduction and motivation

We are living in a time of profound upheaval and change, both socially and technologically, with these two aspects intertwined to a certain extent. From a societal perspective, we are facing the problem of an aging population and the associated challenges in caring for and providing support to our older citizens. According to calculations by the Statistisches Bundesamt (2024), Germany is expected to face a shortage between 280,000 and 690,000 nursing staff by 2049 as a result of the aging society. An aging population requires a new organization and approach to care in order to ensure adequate assistance in care homes, hospitals, and private homes in the future. With increasing age, interpersonal interactions often decline due to changes in social and personal relationships, which can lead to loneliness among older people. Technologies that compensate for the decline in skilled workers and curb skyrocketing costs, particularly in the health and care sectors, could offer a solution here.

Supporting caregivers–who often lack the time for individual social interaction due to staff shortages and heavy workloads–with robots equipped with artificial intelligence, empathy, and powerful cognitive systems offers opportunities to break up the monotonous daily routine in nursing homes. These robots could be used for entertainment purposes to reduce monotony and boredom by bringing personal interaction and conversation into everyday life and fostering social connections, thereby improving the quality of life for older adults. However, this often raises ethical concerns regarding autonomy, social isolation, privacy, and a lack of human communication (Badawy, 2024; Yuan et al., 2023; Chan and Muralidharan, 2024). Essentially, the question arises as to whether additional interaction with machines in the form of social robots in care facilities is better for the wellbeing of residents than social contact with humans alone, which then only takes place rarely. Another fundamental question is whether assistance robots should be introduced as a supplement to caregivers or as a replacement for them. Current knowledge shows that users and nursing staff believe that the use of robots should not be aimed at replacing humans, but rather at taking over certain tasks. This raises the question of the type and scope of tasks that can be delegated to robots and those that should remain the responsibility of humans (Lehoux and Grimard, 2018).

In order for robots to be successfully deployed in healthcare settings, both at home and in healthcare facilities, they must behave in a manner that is appropriate and acceptable to human users. The introduction of robotic assistance in such a sensitive area as a care facility is often accompanied by fears and resistance on the part of staff and older adults (Hebesberger et al., 2017). Tamaki Welply and Lechevalier (2024) examined interpersonal relationships with social robots in nursing homes, focusing on positional and temporal aspects of the relationships between older adults in need of care, their care provider, and the robot. Selecting the right robot and developing the right applications for these specific Human-Robot Interaction (HRI) scenarios is therefore a challenging task, as these robots will not live separately from humans behind safety fences. They will be used in hospitals, healthcare facilities, and in old people’s homes, interacting directly with people who never chose to ‘work with robots’. Klier and Lugrin (2025) proposed five key areas for a structured approach to developing applications for social robots for cognitively healthy older adults: positive emotions, engagement, relationships, meaning, and accomplishment. In short, benefits for older people would be: emotional support, promotion of social interaction, and reduction of loneliness.

In HRI, the ability of robots to meet the demands of their social environment and respond appropriately and comprehensibly is a general requirement (Mahdi et al., 2022). Their understanding of social norms and expectations should guide their actions, and they should respond to the thoughts, feelings, intentions, and appearance of others within a broad social context–in other words, demonstrate *social presence*. In this context, social presence (or co-presence) refers to the sense of being with another person and depends on how easily one feels able to access that person’s intelligence, intentions, and sensory experiences, which we will discuss in more detail in chapter 2.1.

Social presence is considered one of the most important aspects of robots’ social characteristics and has a considerable impact on HRI, since the absence of a distinct sense of social presence reduces the experience with social robots to a purely physical interaction with artificial beings (Fong et al., 2003; Chen N. et al., 2023). Liu et al. (2025) identified social presence as a mediating variable that influences the relationship between the anthropomorphic behavior of service robots and its impact on humans’ desire for diversity. Krämer et al. (2026) presented a theoretical framework for examining the effects of social presence on social cognition and affect in computer-mediated communication and for exploring how mediated interactions shape our understanding of others’ mental states. They explicitly highlight the importance of research in this area for the medical sector, where a personal connection between healthcare providers, caregivers, and patients is crucial to the success of treatment.

Specifically, in our study we examined the extent to which the interplay of certain cognitive abilities–such as language, visual perception, and memories of individual interactions–could alter and, ideally, enhance the perception and effectiveness of such robots as social agents with social presence. We were also interested in whether, during natural-language conversations with a robot on any given topic, additional visual perception of the person and the environment, as well as memories of previous encounters, enhance the naturalness and human-likeness of the interaction. Therefore, this study also examines the degree of *anthropomorphism* associated with these different cognitive abilities of the robot.

## 2 Cognitive abilities in interactive behavior of social robots

We investigated the potential of certain cognitive abilities of a robot for social interaction with humans in terms of perceived social presence and anthropomorphism. To that end, we equipped robots with the following cognitive abilities:

1. Unrestricted natural language capabilities
2. Visual impressions of the interaction partner and the surrounding situation
3. Individual memories of previous interactions

Basically, the question was whether the cognitive abilities of vision and memory had a significant influence on the perception of social presence and the anthropomorphism of the robot, and if so, what that influence was. Since social interactions between humans and machines are only possible if communication can take place according to human-centered standards and habits, the implementation of communication, visual perception, and memory capabilities also had to meet these requirements. In the following, we examine the concept of social presence and discuss approaches to implementing the cognitive abilities of language, vision, and memory for social robots.

### 2.1 Social presence

To explore the question of what kind of robot behavior contributes to its *social presence*, we must first clarify what we mean by *social presence* and distinguish it from related concepts such as *social intelligence* and *social cognition*. Social Presence Theory (SPT) was originally defined by Short et al. as, *the degree of salience of the other person in the interaction and the consequent salience of the interpersonal relationships* (Short et al., 1976). A more modern definition of social presence theory was refined by Gunawardena (1995) to state, *the degree to which a person is perceived as a real person in mediated communication*. Social presence (or co-presence), the feeling of being there with a ‘real’ person, refers to the *sense of being with another* and is a crucial component of interactions and is dependent on the ease with which one perceives to have the *access to the intelligence, intentions, and sensory impressions of another* (Oh et al., 2018; Biocca et al., 2003; Biocca, 1997).

Biocca et al. (2001) examined the factor structure and concurrent validity of the *Networked Minds* measure of social presence and identified eight factors that can be grouped into the areas of *shared presence*, *psychological engagement*, and *behavioral engagement*. The *psychological engagement* area, which includes the factors *mutual attention*, *empathy*, and *mutual understanding*, is of particular interest to our study. The enhanced cognitive abilities in visual perception and memory capabilities that we tested form the basis for shared attention, shared mental models, and predictable behaviors–and thus also for behaviors that may be perceived as empathetic. Konya-Baumbach et al. (2023) found that social presence is also a crucial factor for successful interactions between customers and chatbots.

Social intelligence, as proposed by Thorndike and Stein (1937), is *the ability to understand and manage men and women, boys and girls–to act wisely in human relations*. It can also be interpreted as the ability to understand social situations, interact appropriately, and build harmonious relationships. These skills are a fundamental prerequisite for empathy, situational awareness, and tact, and are necessary for maintaining positive and pleasant relationships with others. de Ruyter et al. (2005) investigated whether a layer of social intelligence in a home dialogue system could elicit positive attitudes towards the technology embedded in the home environment. Their findings confirmed this assumption.

Social Cognition *refers to the abilities of recognizing and controlling the self in relation to others and the abilities of applying and perceiving social signals in interactions with others* (Nagai, 2022). It involves a cognitive coupling between the robot and the human being in the interaction, with a mutual and shared understanding and a common perception of the respective situations and intentions. Brinck and Balkenius (2018) explained that the robot should be susceptible to similar environmental cues and behavior patterns as humans are.

Social presence can manifest itself either on an *ad hoc* basis or over a longer period of time. Leite et al., 2014, 2009 developed an empathy model for social robots interacting with children and examined the social presence of their robots over an extended period. Their findings showed that social presence remained stable over time. In the context of interactions between children and robots, Chen Y.-C. et al. (2023) also found that the perception of a robot’s social presence is positively correlated with an improved relationship with that robot. Their findings showed that children who attributed a higher level of social presence to the robot formed a stronger bond with it, underscoring the importance of robots’ social presence in fostering positive relationships during interactions between children and robots. Jung et al. (2004) conducted experiments to investigate the relative effectiveness of physical embodiment on the social presence of social robots. Their results showed that the physical embodiment of social robots had a positive effect on the sense of social presence, particularly when participants were able to touch the robot. Chang et al. (2025) noted that excessive empathy or an overly pronounced social presence could cause a robot to communicate in a more emotional manner than might be intended by an higher-level objective.

Even though robots cannot really be considered moral actors with their own beliefs, desires, and intentions, and therefore cannot actually be objects of trust, it is important to note that humans tend to treat them as if they were (Vinanzi, 2025). Human-like characteristics in a robot lead to *anthropomorphism*. This phenomenon is considered desirable in social robots, as it supports their functionality and improves their sociality. Lee et al. (2006) found that humans are able to accurately recognize a robot’s personality based on its verbal and nonverbal behaviors. Interestingly, their research showed that people enjoyed interacting with a robot more when its personality (introverted vs. extroverted) complemented their own personality than when the robot’s personality resembled their own. The social presence perceived by participants during interaction proved to be an important mediator for the observed complementary attraction.

### 2.2 LLMs for dialogue and cognition

The use of Large Language Models (LLMs), for example, from OpenAI (2026) or the European Mistral AI (2026), via their Application Programming Interface (API) to generate comprehensive language skills for robots has been the subject of current research for some time. Using LLMs allows not only Text to Speech (TTS) and Speech to Text (STT) but also reasoning and judgment approaches that can be used for robot responses (Sievers, 2025). Vision Language Models (VLMs) supplement these capabilities with a sense of sight, enabling the robot to reason and talk about visual impressions in natural language (Ghosh et al., 2024). These visual aspects are discussed in more detail in chapter 2.3. For our study, we chose Mistral AI’s language models because an LLM hosted in Europe offers European users a solution that complies with European data protection regulations.

With this successes of language models in various fields, interest in the interplay between LLMs and cognitive architectures has also increased, and ways of integrating LLMs with cognitive architectures have been discussed (Dubey et al., 2024). Insights from human cognition and psychology that underlie cognitive architectures can contribute to the development of LLM-based systems that are more efficient, reliable, and human-like (Sun, 2024). Although LLMs are in principle capable of making quick automatic inferences, they do not possess the high-level cognitive abilities required for complex operations and ‘slow thinking’, as described in the dual process theory of human cognition (Kahneman, 2011). To overcome this limitation, Knowles et al. (2024) proposed an architecture that combines LLMs and cognitive architectures. Niu et al. (2024) provided an overview of similarities, differences and challenges in their review on LLMs and cognitive science by analyzing methods to assess possible cognitive capabilities of LLMs, discussing biases and limitations as well as an integration of LLMs with cognitive architectures. Binz and Schulz (2024) investigated whether LLMs can be used as cognitive models after fine-tuning them with data from psychological experiments. They found that these models provided accurate representations of human behavior and were also able to predict human performance in a previously unseen task.

One risk associated with using LLMs to generate responses is the danger of so-called *hallucinations*, i.e., incorrect results generated by LLMs that are not based on facts. Providing factual information relevant to the current interaction–but unknown to the LLM–as prompt information for the language model can help to at least reduce such missteps. An understanding of the current situation and supplementary facts, e.g., from memory, delivered via the system prompt, can clarify the facts for the LLM to such an extent that accurate responses are possible even in dynamic settings for which the LLM has no prior knowledge.

### 2.3 Vision as a foundation for common ground

With the rise of LLMs and their success in HRI applications, communicating with robots in natural language has become easy and almost normal. But language alone sometimes does not convey everything that is important in a dialogue. Ambiguities and allusions can often only be resolved if visual information is also available. Especially for the deployment of social robots in everyday situations, such as in domestic environments or in care settings, and for uncomplicated, natural use, it is essential that the robot has an understanding that is appropriate to human customs. Robots for social interactions should be designed and developed from a human-centered perspective so that they can meet the requirements of their social environment and respond appropriately and comprehensibly. Social norms and expectations should guide the robots’ decision-making, and the thoughts and intentions of their human counterparts should be understood in a broader social context. The inclusion of visual information helps to correctly classify the context during such an interaction. It should be noted that VLMs are also capable of reading text in their field of vision, e.g., on clothing or pictures on the wall.

Depending on the specific task, there are other ways besides VLMs to equip a service robot with visual perception capabilities. Trinquet et al. (2025) integrated the robot’s camera with computer vision techniques for a specific scenario in a library, enabling the robot to identify the books in front of it and read their titles. To do this, they employed object recognition models and Optical Character Recognition (OCR) models. An application for improving traditional text-based prompts for LLMs with real-time visual inputs was presented by Abbo and Belpaeme (2025). The text from dialogues between people in various settings and a robot was supplemented with summaries of images from the robot’s perspective for prompt engineering. Images from a video recorded during the conversation were used as visual input.

### 2.4 Recollections for social interactions

A central question of AI-driven cognitive robotics is how knowledge and experience can be efficiently organized and managed in a robot control architecture. A memory system is seen as a central component of such a control architecture for high-level cognitive abilities such as reasoning and prospection (Peller-Konrad et al., 2023). And LLMs alone are not sufficient for human-robot communication, since robots must base their responses on their understanding of the specific situation in which they find themselves. Memory therefore becomes the key component of an agents cognitive system in order to incorporate situational knowledge about the world into communication (Silva et al., 2023). Decision-making is a highly cognitive task based on cognitive processes such as perception, attention, and memory. In real-world situations, appropriate strategies must be found and a series of decisions made, with each decision dependent on feedback from a potentially changing environment. The underlying processes of dynamic decision-making can be investigated using the method of cognitive modeling (Prezenski et al., 2017).

LLMs are by nature stateless, meaning they cannot store information across interaction turns (Yu et al., 2025). When LLMs are used in typical multi-turn conversations, in addition to each request from the serving system, a growing log of the conversation history must be processed at each conversation turn. People do not try to remember everything, but optimize their memory by selecting what they remember and what they do not. This is where the principle of bounded rationality comes into effect, allowing us to make ‘good enough’ decisions under cognitive constraints (Hertwig and Todd, 2003). We as humans tend to use heuristics to arrive at probable conclusions or practical solutions based on educated guesses, despite limited knowledge and time constraints.

Various methods have already been tested to implement long-term memory for personalized interactions with the interaction partner in HRI. For example, Paplu et al. (2022) used a MySQL database to store memory content. Shahabian Alashti et al. (2026) proposed an architecture that incorporates memory-based thinking and context awareness and includes, among other things, a long-term memory for experiential knowledge. A query answering system for healthcare assistance of elderly storing preprocessed data into a database and giving long term memory to robots was introduced by Idrees et al. (2020). Sánchez et al. (2015) proposed a framework for providing a robot with episodic long-term memory, giving it the ability to incorporate past experiences when interacting with humans. Saladino et al. (2026) proposed a cognitive architecture for HRI that leverages the common sense logic embedded in LLMs to handle dynamic scenarios, including a long-term memory that can be accessed via Retrieval-Augmented Generation (RAG) techniques. The use of RAG can help reduce hallucinating–i.e., inventing alleged facts–when an LLM lacks factual knowledge (Gao et al., 2024; Sanmartin, 2024). RAG approaches combine and optimize external sources in LLMs to integrate relevant knowledge, but cause significant computational load and latency during inference due to the complex search for nearest neighbors in large data stores and extended context (Cao et al., 2025).

Sumers et al. (2024) proposed a language agent with a framework for modular memory components and a generalized decision making process that combines insights from symbolic artificial intelligence and cognitive science. As some form of effective memory is required to produce semantically consistent text, LLMs were also tested for their memory capabilities (Janik, 2024). An examination of the memory properties of LLMs revealed similarities with important features of human memory, but in the case of an LLM these are learned from the statistics of the training text data rather than from the architecture of the LLM. The question of whether the current generation of LLMs is able to develop grounded cognition that incorporates prior expectations and prior world experiences to see the bigger picture was analyzed by Leivada et al. (2024), but their conclusions remained rather skeptical and corresponding abilities of LLMs can be considered rather premature.

In addition, there are already various approaches to using the Adaptive Control of Thought-Rational (ACT-R) cognitive architecture to implement memory functions (Anderson et al., 2004). ACT-R, for example, has been combined with LLMs to enable human-centered decision-making by integrating knowledge from the cognitive model’s decision-making process as neural representations into trainable layers of the LLM (Wu et al., 2024). Honda et al. (2026) proposed an approach to implement a human-like memory mechanism based on ACT-R with LLM integration, replacing the chunk activation from base-level learning typical of ACT-R with a vector-based activation mechanism and calculating the cosine similarity between a memory search query and each chunk. For cognitive models that use spreading activation, in which connections are evaluated according to associations as in ACT-R, Meghdadi et al. (2026) proposed the use of association metrics derived from language model embeddings to create scalable models for associative priming for a lexical decision task. This approach also served as proof of concept for the integration of embedding-based representations into algorithmic models of language processing.

#### 2.4.1 ACT-R and memory

Cognitive architectures such as ACT-R attempt to describe and integrate the basic mechanisms of human cognition. They refer both to a theory about the structure of the human mind and to a software-based implementation of such a theory. In doing so, they rely on empirically supported assumptions from cognitive psychology. Their formalized models can be used to react flexibly to actions in a human-like manner and to develop a situational understanding regarding human behavior for adequate reactions. They are particularly suitable for human cognitive modeling (Kurup and Lebiere, 2012). In previous work, we have already used the ACT-R cognitive architecture in conjunction with a social robot to store and process individual memories (Sievers and Russwinkel, 2026; 2025).

ACT-R comprises a declarative and a procedural memory, whereby the declarative memory supports lexical knowledge by encoding, storing and retrieving semantic knowledge, as in humans, while the procedural memory enables the learning of habits and skills (Whitehill, 2013). The procedural memory module functions as a production system in which procedural knowledge from production rules triggers an action when certain conditions are met. The declarative memory module stores knowledge in the form of slot-value pairs known as chunks. A slot is a feature or attribute that is associated with a value like (name, thomas). Formally, the slot-value pairs of chunk *n* are represented as ${\text{c}}_{n}$ = [( ${\text{s}}_{n1}$ , ${\text{v}}_{n1}$ ), … ( ${\text{s}}_{nS}$ , ${\text{v}}_{nS}$ ),], where *s* is the slot, *v* is the associated value and *S* is the number of pairs. Cognition arises in ACT-R via a series of production cycles that involve the selection and execution of production rules. A production rule is only selected if the buffer contents are an exact match to the selection criteria. Nevertheless, this restriction can be relaxed to allow for procedural errors and stochastic behavior. Once a production rule is selected, the actions specified in the rule are executed, changing the contents of some buffers, which in turn allows another production rule to be selected in the next production cycle. The strength of the recollection and thus the probability of selecting this particular chunk is referred to as *activation*. A chunk can gain activation by using it. To do so, it must match a production rule that is triggered. ACT-R is available as a software package for installation and use via the ‘Lisp’ programming language or as an implementation in Python supporting symbolic and sub-symbolic processes and covering all basic cases of ACT-R modeling (Carnegie Mellon University (2024); Brasoveanu and Dotlacil (2020); Jakdot (2026).

## 3 Methods

The aim of this study was to investigate the influence of various cognitive abilities of a robot on how robots are perceived and evaluated in HRI, particularly with regard to their *social presence* and *anthropomorphism*. The different cognitive abilities to be examined were as follows:

1. Unrestricted natural language capabilities
2. Visual impressions of the interaction partner and the surrounding situation
3. Individual memories of previous interactions

Study participants were asked to engage in casual, brief conversations with two robots. In principle, the topics discussed during the dialogue could be anything. The robot’s additional ability to visually perceive its human interaction partner and their surroundings enabled it to incorporate aspects of this perception into the conversation. Otherwise, the robot was able to recall the content of a previous conversation and thus share personalized information with the person it was talking to. After receiving general information about the study and the robots, the participants engaged in conversations with the robots and completed two short questionnaires following each conversation. The participants were not informed in advance about the robots’ different capabilities. The Ethics Committee at the University of Lübeck has approved this study. There were a total of four rounds of dialogic interaction. The first two rounds consisted of introductory conversations with two different robots, both of which relied solely on “speaking” as their ability. This was followed by another conversation with each robot over the next two rounds. There were two different conditions involving different additional abilities of the robot:

- Condition A: “Speech + Vision” (Robot A)
- Condition B: “Speech + Memory” (Robot B)

The study was conducted in a within-subjects design, meaning that each participant completes all four trials with both conditions, with the order of conditions (here, robots) A and B randomized for each participant; that is, some participants met Robot A first, while others met Robot B first. This results in the following example procedure for a participant:

1. Trial: “Speaking” (introductory conversation with Robot A)
2. Trial: “Speaking” (introductory conversation with Robot B)
3. Trial: “Speaking + Seeing” (Robot A)
4. Trial: “Speaking + Memory” (Robot B)

After each of the four conversations, the participant was asked to complete two questionnaires:

1. Seven questions on social presence using a five-point Likert scale (strongly disagree, somewhat disagree, neutral, somewhat agree, strongly agree).
2. The Human-Robot Interaction Evaluation Scale (HRIES) questionnaire consisting of 16 items, each rated on a numerical scale from 1 (not at all) to 7 (completely) assessing the four anthropomorphic dimensions of *sociability*, *animacy*, *agency*, and *disturbance.*

### 3.1 Hypotheses

In line with the research in Čaić et al. (2020), we assumed that a demonstration of more human-like abilities would be associated with a greater sense of social presence. Our hypotheses regarding the effect of the examined different additional cognitive abilities on the perception of social presence were as follows:

Hypothesis 1 A robot that visually perceives its conversation partner and their surroundings and incorporates details from that perception into the conversation is attributed with increased social presence.

Hypothesis 2 A robot that remembers specific details from a previous conversation and incorporates them into a subsequent conversation is also considered to have a greater social presence.

We also expected that, for both types of cognitive enhancement, certain characteristics related to the anthropomorphic dimensions identified by HRIES would change.

### 3.2 Participants

Study participants were recruited through a Moodle forum for studies at the University of Lübeck and by approaching them in person. The only eligibility criterion was sufficient proficiency in German to be able to conduct the dialogs in German. A total of 32 participants aged between 19 and 60 took part in this study, which was based on a within-subjects design. Of these, 19 were female and 13 were male. No one identified as non-binary, and the average age was 27 years, with a standard deviation of 8.75. The female participants were predominantly from the fields of psychology, medicine, or nursing, while the male participants mostly had a technical or computer science background. Professional background and areas of interest resulted from the circumstances at our university and the participants’ willingness to take part in this study. With the exception of three male participants, none of the others had any prior experience with social robots.

The study took place in a room at our institute. Participants were not required to provide any personal information other than their first name, which could even be fictitious. The name was needed by the robot with memory function to recall details from the previous conversation with the participant during the second conversation.

### 3.3 Experimental Setup

We had two Pepper robots that could be distinguished from one another by their names and their outfits (see Figure 1):

- Robot “Charlie” for an introductory conversation and for the ability to perceive visual impressions in a second interaction.
- Robot “Kim” for another introductory conversation and for the memory function in a second interaction.

**FIGURE 1:**
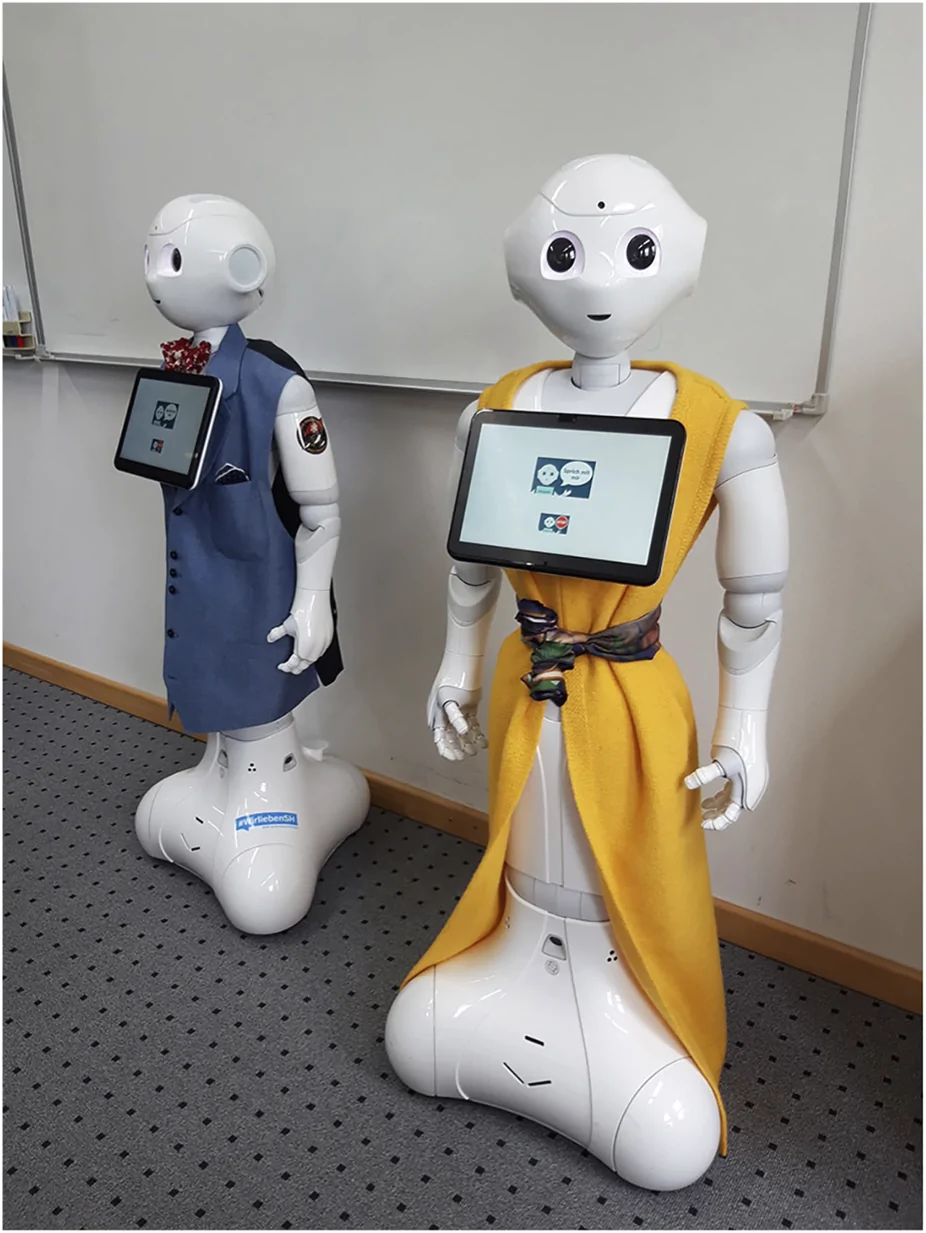
Robots Charlie and Kim dressed for service.

The robots used a large language model from Mistral AI to generate responses, which runs on European servers in compliance with the GDPR. The memory chunks were stored on the experimenter’s web server via an SSL-encrypted connection for the duration of the experiment and were subsequently deleted. Images from the robot camera and content data were not stored beyond the duration of the conversation. Prompts for the system role instructed the LLM to execute the tasks as a completion task using zero-shot prompting Brown et al. (2020). All dialogues were conducted in German and the prompts for the LLM/VLM were also given in German.

The ACT-R cognitive model, which was implemented in our robot applications for this study using the Python implementation *pyactr*, was provided with chunks containing the speaker’s name and keywords for memory association. The actual recollection phrase was stored in a slot within such a memory chunk, from which it could be retrieved. The keywords and phrases were generated by the LLM from conversation content. The model had to check whether there were already any memory chunks with this same name indicating an existing recollection for this person. If this was the case, the model searched for matching chunks already stored for the specified keywords. For simplicity reasons the transfer of not more than three keywords was assumed. The model’s productions of the procedural memory checked all combinations of the keyword sequence for matches with the memory content, generated a hit based on similarity and chunk activation, and retrieved the corresponding recollection phrase. This recollection was then used to augment the LLM prompt so that the language model could refer to it during text generation. New memories were stored as chunks in declarative memory after each dialogue turn.

The Mistral multimodal model Pixtral 12B (pixtral-12b-2409) was used for text and image processing in our study (Agrawal et al., 2024). The VLM was instructed to relate to the content of an image taken with the robot’s camera equipment. The robot’s front camera took a picture every 4 seconds to capture an up-to-date impression of what the robot saw. The interval of 4 seconds was chosen arbitrarily, but seemed to be a practical compromise between timeliness and resource efficiency. The latest image was sent to the API of the model for chat completion together with the person’s statement. Figure 2 shows a schematic representation of the application for the Pepper robots, which connected to the LLM or VLM via API, as well as the integration with the ACT-R model for memory, which ran on a web server and was also connected online.

**FIGURE 2:**
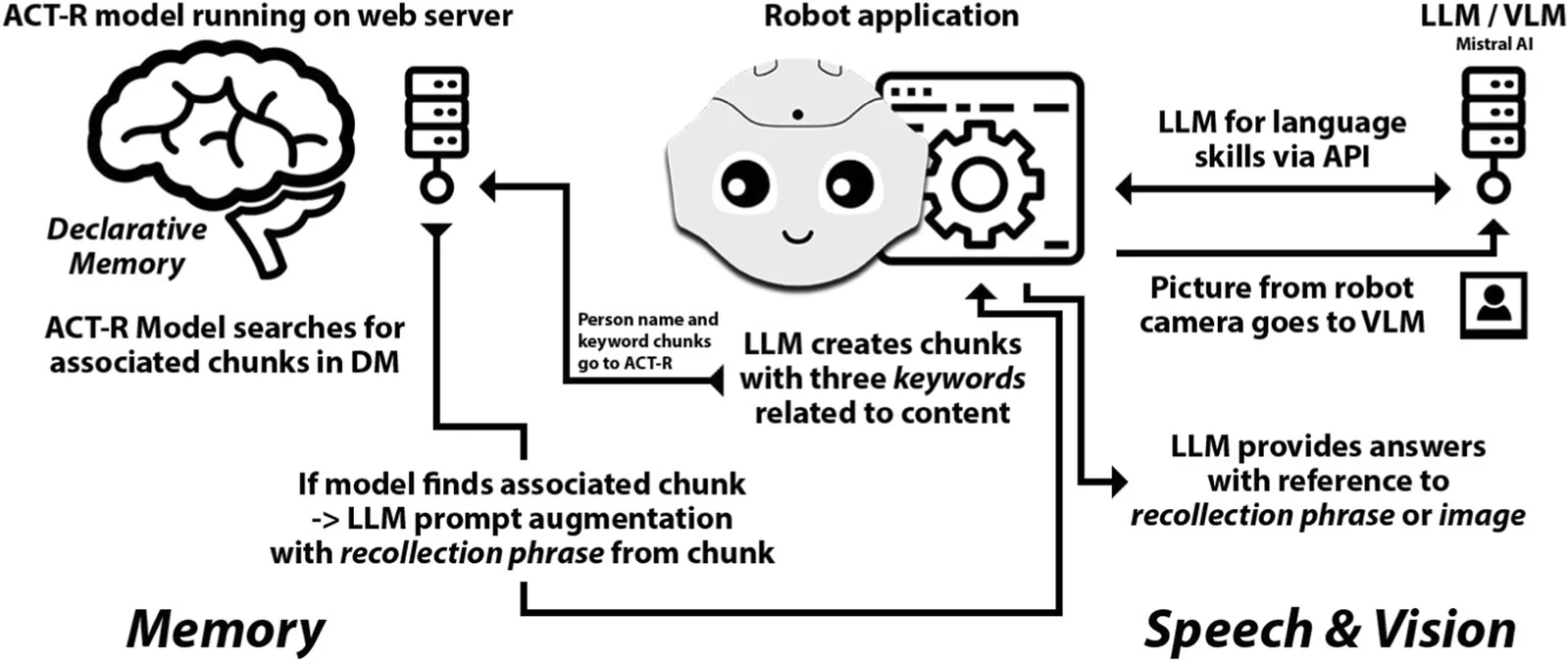
Schematic representation of the application for the Pepper robot, incorporating the LLM or VLM and the ACT-R model for memory.

In all conversations, the robots attempted to discuss the topic of fashion with their conversation partners. Each robot suggested this topic to provide a conversation starter, but it was irrelevant to the study; any other topic could be discussed instead. The Pepper robots accompanied their remarks with random movements and gestures. Among other things, the conversation partner was asked to evaluate the robot’s appearance. If the robot recalled a previous conversation, the system prompt for the LLM instructed it to address that recollection. When visual information was used, the system prompt instructed the LLM to incorporate that information–including details about the conversation partner, any accessories they might be wearing, and information about the surroundings–into the conversation. A conversation round lasted approximately 2–3 min. The order of the robots was varied in terms of both cognitive abilities and clothing to rule out any potential order effects. The study’s procedure regarding the order of the activities could, for example, look like this:

1. Introductory Conversation 1: Robot “Charlie” introduces itself, asks the participant’s first name, and starts a conversation about fashion.
2. Introductory Conversation 2: Robot “Kim” introduces itself, asks the participant’s first name, and also starts a conversation about fashion.
3. Robot “Charlie” incorporates visual impressions of the conversation partner and the surroundings (e.g., clothing, accessories …) into the second conversation.
4. Robot “Kim” engages in a second conversation, during which it may refer to details from the initial conversation that it has remembered.

### 3.4 Data collection

There are various ways to measure a robot’s *social presence* during interaction. Chen N. et al. (2023), for example, created a theoretical model of the social presence of robots and developed corresponding measurement scales. However, we decided to adopt a questionnaire consisting of seven questions using a five-point Likert scale from 1 = *strongly disagree* to 5 = *strongly agree*, which was adapted from studies conducted by Heerink et al. (2010a) and Lee et al. 2006, 2005, and to modify it to fit the present study. The questionnaire was written in German; The English equivalents of the questions are as follows:

1. I felt like I was talking to a real person
2. I felt like the robot was really paying attention to me
3. I felt like the robot was responding to me personally
4. I felt like someone was really listening to me
5. I felt like I was dealing with a living being
6. I felt that there was a certain personal connection
7. I found the conversation captivating

To assess *anthropomorphism*, we used the German translation of the Human-Robot Interaction Evaluation Scale (HRIES) according to Kühnlenz and Kühnlenz (2025) as our second questionnaire. HRIES is a rigorously validated questionnaire designed to assess the anthropomorphic dimensions of *sociability*, *animacy*, *agency*, and *disturbance*, with each dimension consisting of four items. It measures agreement with the robot stimulus on a seven-point Likert scale from 1 (not at all) to 7 (completely), has been used in numerous studies on human-robot interaction, and is characterized by its high reliability. The experimenter was present during the conversations as an observer.

### 3.5 Data analysis

To analyze the questionnaire data on social presence and anthropomorphism, we calculated the average scores for all participants. The range of scores for social presence is from 1 to 5, based on the five-point Likert scale used here. Each of the four HRIES anthropomorphic dimensions sociability, animacy, agency, and disturbance consists of four different items. The lowest possible score for each dimension on the seven-point Likert scale is therefore 4, and the highest possible score is 28.

In order to assess the significance of our results, the Wilcoxon signed-rank test was used instead of a paired t-test, since the differences between all our measurements were not normally distributed and thus did not meet the assumptions required for a parametric test. Normality was assessed primarily using Q-Q plots, with the Shapiro-Wilk test used as a supplementary measure. In addition, we applied the Holm-Bonferroni method for adjusting multiple tests in order to control the family-wise error rate across all the different significance tests. We used an alpha level of 0.05 for all statistical tests.

## 4 Results

Following adjustment for multiple testing using the Holm-Bonferroni procedure to control the family-wise error rate across 22 significance tests (7 questions regarding social presence plus four anthropomorphic dimensions for each of the two robots), only two variables remained statistically significant: the social presence question “the robot was really paying attention to me” for robot “Charlie” (Wilcoxon signed-rank test, p < 0.05) and the anthropomorphic dimension *disturbance* for robot “Kim” (Wilcoxon signed-rank test, p < 0.05). Given the limited sample size and consequently reduced statistical power, these results should be interpreted cautiously. In particular, the correction for multiple comparisons, while reducing the likelihood of false-positive findings, further increases the conservativeness of the analysis and may have contributed to an increased risk of false-negative results. Therefore, non-significant findings after correction should not be taken as definitive evidence of the absence of effects.

### 4.1 Hypothesis 1

Figure 3 shows the overall average score for all seven *social presence* questions from the first round with robot “Charlie”, in which the robot can only speak, and the perception of *social presence* when the robot in the second round can also visually perceive the conversation partner and the environment and incorporates details from this perception–e.g., regarding the conversation partner’s clothing, accessories, or general appearance–into the conversation. The overall average score for all seven questions, as well as standard deviations, for the social presence of the robot “Charlie” is also shown in table Table 1.

**FIGURE 3:**
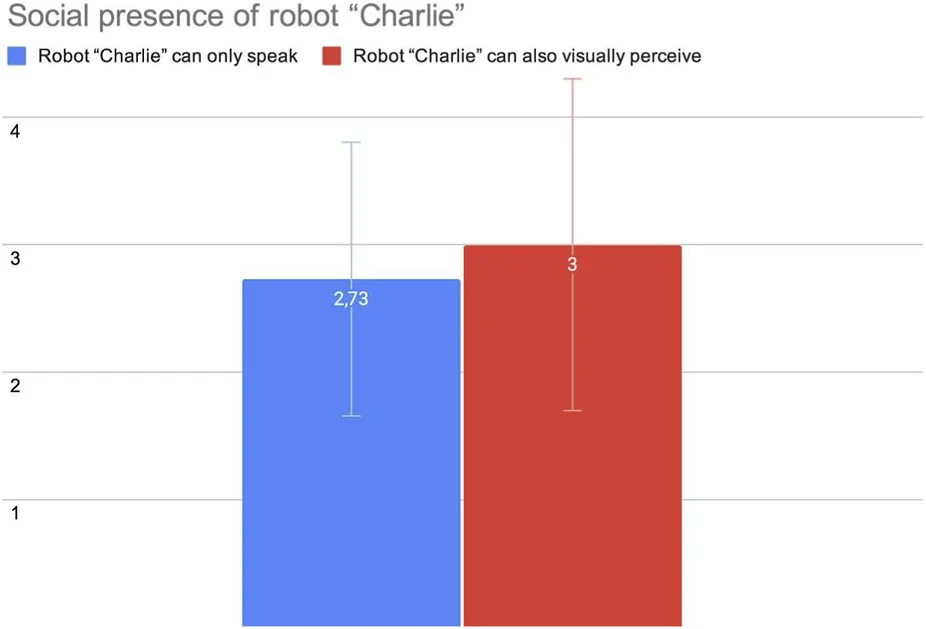
Evaluation results showing average scores for the social presence of the robot “Charlie” on a five-point Likert scale (1 = strongly disagree, 5 = strongly agree). Error bars show standard deviations.

**TABLE 1:** Overall average and standard deviation for the social presence of the robot “Charlie”.

Robot capabilities	M	SD
Robot “Charlie” can only speak	2.73	1.08
Robot “Charlie” can also visually perceive	3.0	1.30

We found that considerably higher scores were achieved in the second round, particularly for questions 2 (*paying attention*), 3 (*responds to me*), 5 (*dealing with a living being*), and 7 (*conversation was captivating*). However, the p-values received by the Holm-Bonferroni procedure used for multiple testing to compare the questions between the first and second rounds showed mostly no statistically significant difference, with one exception. There was a significant increase regarding question 2 (*paying attention*), the p-value of the Wilcoxon signed-rank test was 0.046, which is below the significance level of 0.05 and is therefore statistically significant. Table 2 shows the average results for the individual questions regarding the social presence of the robot “Charlie” for the two conditions, including standard deviations SD (baseline) and SD (visual perception), as well as the p-values for the Wilcoxon signed-rank test and the Holm-Bonferroni method.

**TABLE 2:** Results for the individual questions regarding the social presence of the robot “Charlie” with standard deviations and p-values for the Wilcoxon signed-rank test and Holm-Bonferroni.

Question	M (baseline)	M (visual perception)	SD (baseline)	SD (vis. Perception)	Wilcoxon	Holm-Bonf
1: Real person	2.22	2.41	0.79	1.24	0.470	1.0
2: Paying attention	3.13	3.84	1.07	1.14	0.002	0.046
3: Responds to me	3.41	3.84	0.80	1.22	0.034	0.685
4: Listens to me	3.06	3.09	0.88	1.17	0.812	1.0
5: Living being	2.0	2.31	0.98	1.12	0.176	1.0
6: Personal connection	2.53	2.44	1.05	1.08	0.749	1.0
7: Captivating conversation	2.78	3.03	1.21	1.20	0.258	1.0

With regard to the HRIES anthropomorphic dimensions, only a slightly higher value for *disturbance* and a small decrease for *sociability* could be observed here; however, these were not statistically significant (see Figures 4a, b). Table 3 shows the results for the HRIES anthropomorphic dimensions of the robot “Charlie” for the two conditions, including standard deviations SD (baseline) and SD (visual perception), as well as the p-values for the Wilcoxon signed-rank test and the Holm-Bonferroni method. Thus, our hypothesis regarding increased social presence has been confirmed to a certain extent in this case, while only minor changes were observed in the anthropomorphic dimensions, without statistical significance.

**FIGURE 4:**
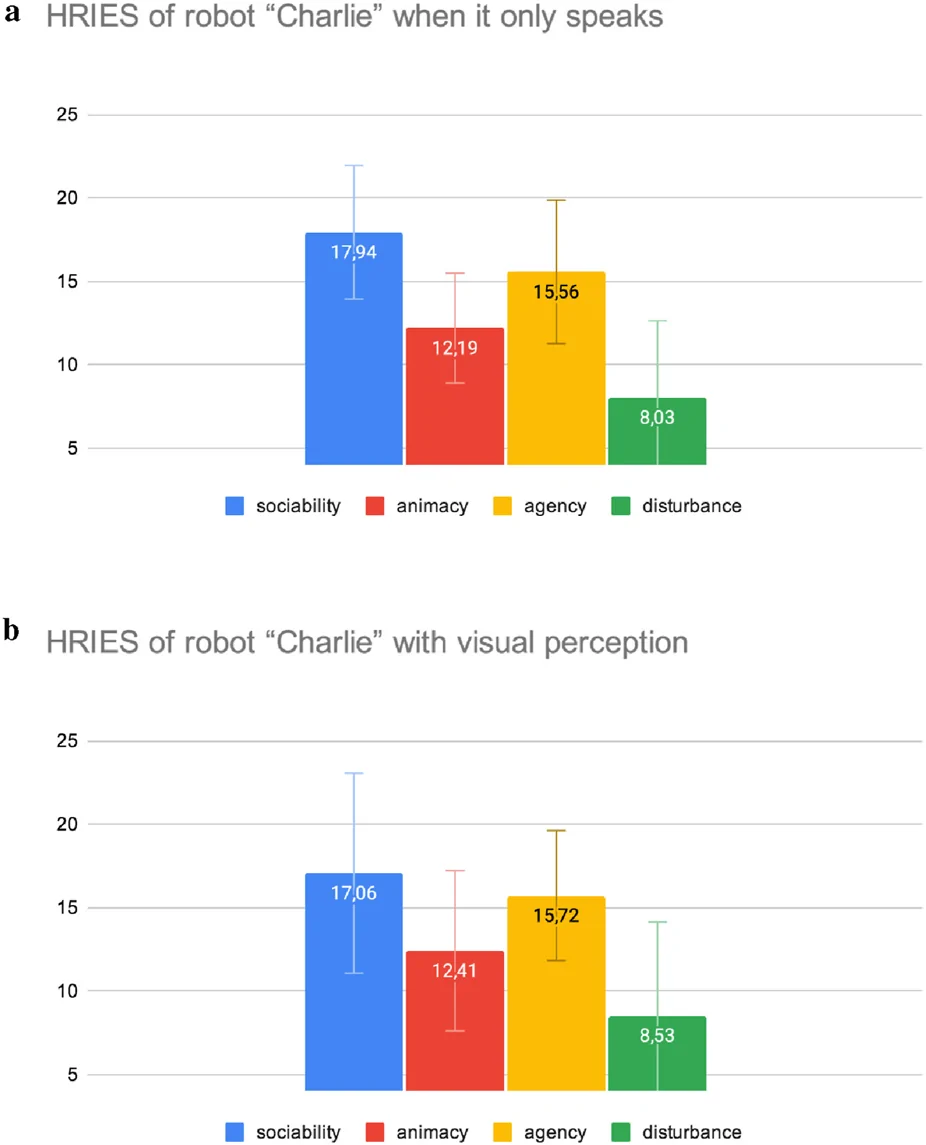
Evaluation results regarding the HRIES anthropomorphic dimensions of the robot “Charlie” with average values for each dimension. The theoretical value range of the seven-point Likert scale, with four items per dimension, lies between four and 28. Error bars show standard deviations. **(a)** HRIES anthropomorphic dimensions of the robot “Charlie” when it relies solely on its speech capabilities. **(b)** HRIES anthropomorphic dimensions of the robot “Charlie” with added visual capabilities.

**TABLE 3:** Results for the HRIES anthropomorphic dimensions of the robot “Charlie” with standard deviations and p-values for the Wilcoxon signed-rank test and Holm-Bonferroni.

Dimension	M (baseline)	M (visual perception)	SD (baseline)	SD (visual perception)	Wilcoxon	Holm-Bonf
Sociability	17.94	17.06	4.6	6.0	0.249	1.0
Animacy	12.19	12.41	4.0	4.8	0.828	1.0
Agency	15.56	15.72	3.3	3.9	0.585	1.0
Disturbance	8.03	8.53	4.3	5.6	0.530	1.0

### 4.2 Hypothesis 2

The assessments of social presence with and without memory, when using the robot “Kim”, were very similar (see Figure 5 and table Table 4 for the overall average score for all seven *social presence* questions). Table 5 shows the results for the individual questions regarding the social presence of the robot “Kim” for the two conditions, including standard deviations SD (baseline) and SD (memory), as well as the p-values for the Wilcoxon signed-rank test and the Holm-Bonferroni method. The Wilcoxon signed-rank test yielded a p-value of less than 0.05 for question 1, but according to the Holm-Bonferroni correction, no statistical significance was found here.

**FIGURE 5:**
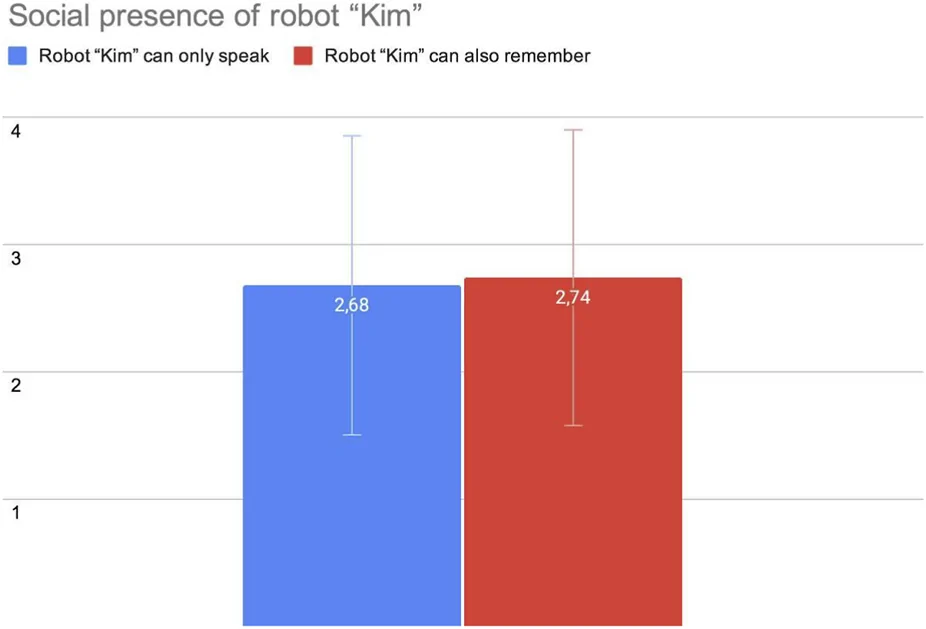
Evaluation results showing average scores for the social presence of the robot “Kim” on a five-point Likert scale (1 = strongly disagree, 5 = strongly agree). Error bars show standard deviations.

**TABLE 4:** Overall average and standard deviation for the social presence of the robot “Kim”.

Robot capabilities	M	SD
Robot “Kim” can only speak	2.68	1.18
Robot “Kim” can also remember	2.74	1.16

**TABLE 5:** Results for the individual questions regarding the social presence of the robot “Kim” with standard deviations and p-values for the Wilcoxon signed-rank test and Holm-Bonferroni.

Question	M (baseline)	M (memory)	SD (baseline)	SD (memory)	Wilcoxon	Holm-Bonf
1: Real person	2.22	2.53	1.01	1.16	0.045	1.0
2: Paying attention	3.09	3.09	0.96	1.15	1.0	1.0
3: Responds to me	3.53	3.34	1.14	0.97	0.313	1.0
4: Listens to me	3.0	3.09	1.02	1.15	0.749	1.0
5: Living being	1.84	2.0	0.85	0.98	0.180	1.0
6: Personal connection	2.31	2.28	1.15	0.99	1.0	1.0
7: Captivating conversation	2.78	2.84	1.24	1.67	0.508	1.0

The HRIES measurements in contrary showed a statistically significant decrease in *disturbance* for the second round, but otherwise there were hardly any differences (see Figures 6a, b). Table 6 shows the average results for the HRIES anthropomorphic dimensions of the robot “Kim” for the two conditions, including standard deviations SD (baseline) and SD (memory), as well as the p-values for the Wilcoxon signed-rank test and the Holm-Bonferroni method. In this case, our hypothesis regarding increased social presence was not confirmed, but the ability of the robot to remember details from a previous conversation appears to reduce the anthropomorphic dimension of disturbance.

**FIGURE 6:**
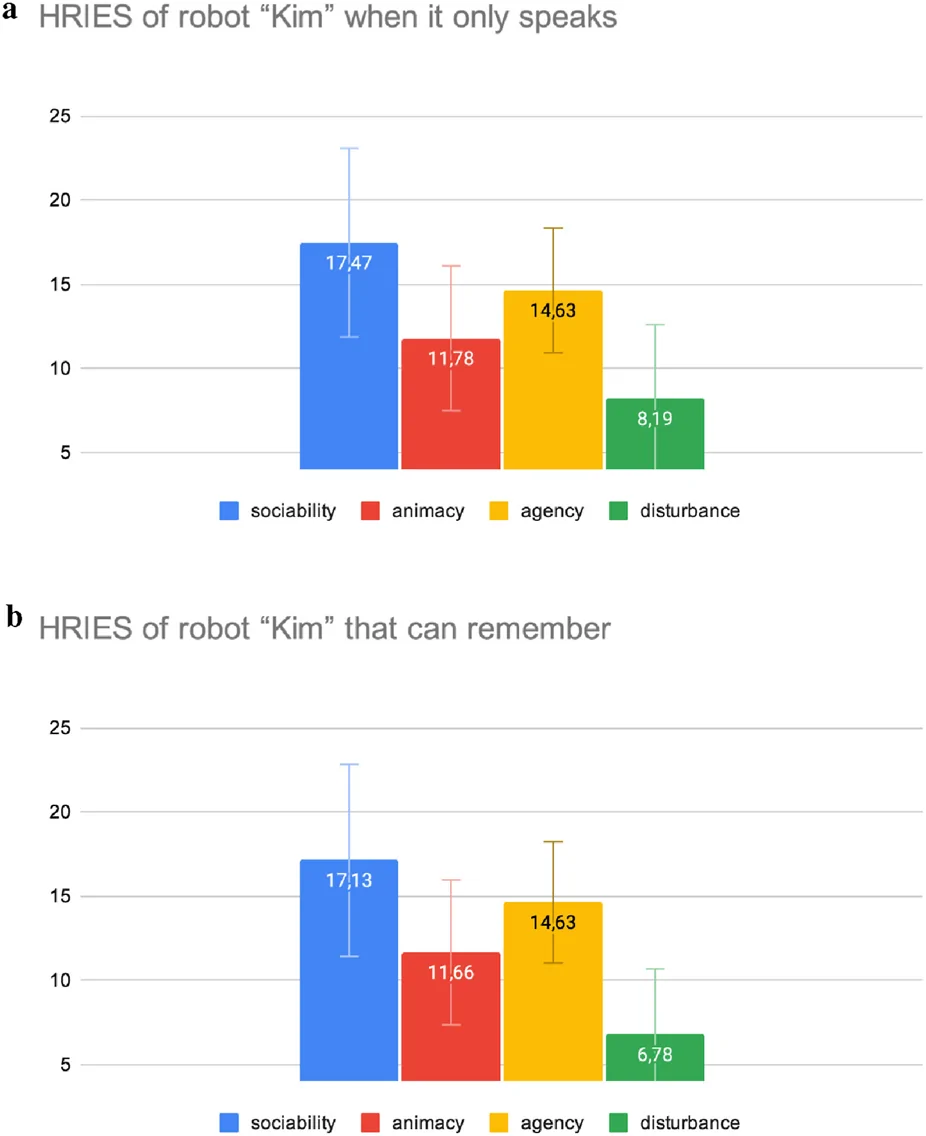
Evaluation results regarding the HRIES anthropomorphic dimensions of the robot “Kim” with average values for each dimension. The theoretical value range of the seven-point Likert scale, with four items per dimension, lies between four and 28. Error bars show standard deviations. **(a)** HRIES anthropomorphic dimensions of the robot “Kim” when it relies solely on its speech capabilities. **(b)** HRIES anthropomorphic dimensions of the robot “Kim” with added memory capabilities.

**TABLE 6:** Results for the HRIES anthropomorphic dimensions of the robot “Kim” with standard deviations and p-values for the Wilcoxon signed-rank test and Holm-Bonferroni.

Dimension	M (baseline)	M (memory)	SD (baseline)	SD (memory)	Wilcoxon	Holm-Bonf
Sociability	17.47	17.13	5.6	5.7	0.409	1.0
Animacy	11.78	11.66	4.3	4.3	0.820	1.0
Agency	14.63	14.63	3.7	3.6	0.973	1.0
Disturbance	8.19	6.78	4.4	3.9	<0.001	0.001

### 4.3 Analysis by gender

A closer look at the analyses of social presence and anthropomorphism according to HRIES reveals gender-specific differences. In almost all cases, the scores for male participants were higher than those for female participants, both in terms of social presence and the anthropomorphic dimensions of HRIES. With regard to social presence, trends toward consistently higher scores among male participants were observed in terms of being perceived as a *real person*, *personal connection*, and *captivating conversation*. As far as the HRIES anthropomorphic dimensions are concerned, what stands out in particular is a considerably higher level of *sociability*, *animacy*, and *agency* among male participants across all of the conversations (see figure Figure 7 and figure Figure 8). Only the dimension of disturbance is lower in both conditions with the robot “Charlie” than it is among the female participants. One explanation for these differences could be the participants’ varying professional backgrounds and, consequently, their differing attitudes and interests.

**FIGURE 7:**
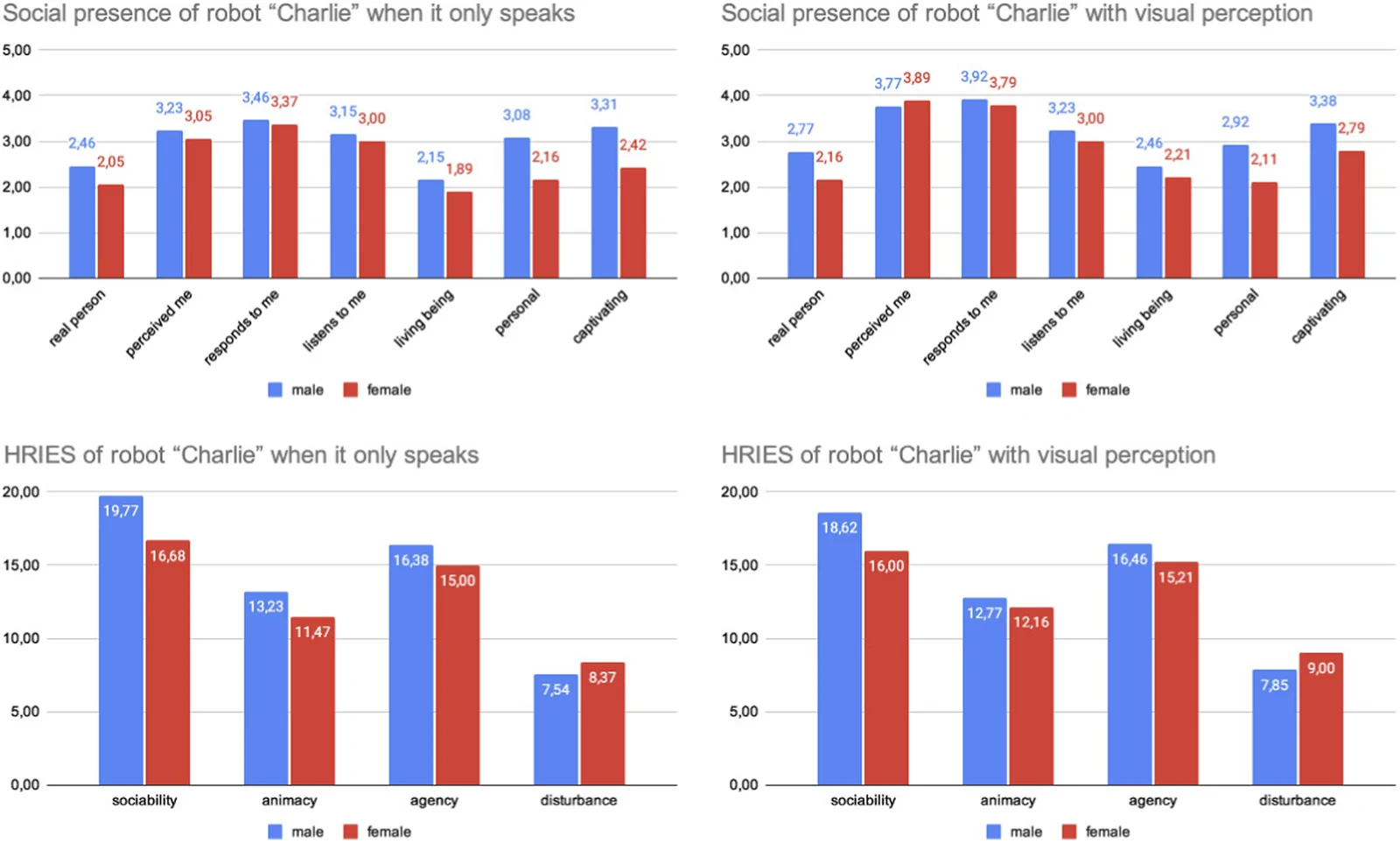
Evaluation results of the robot “Charlie” by gender: Social presence and HRIES anthropomorphic dimensions.

**FIGURE 8:**
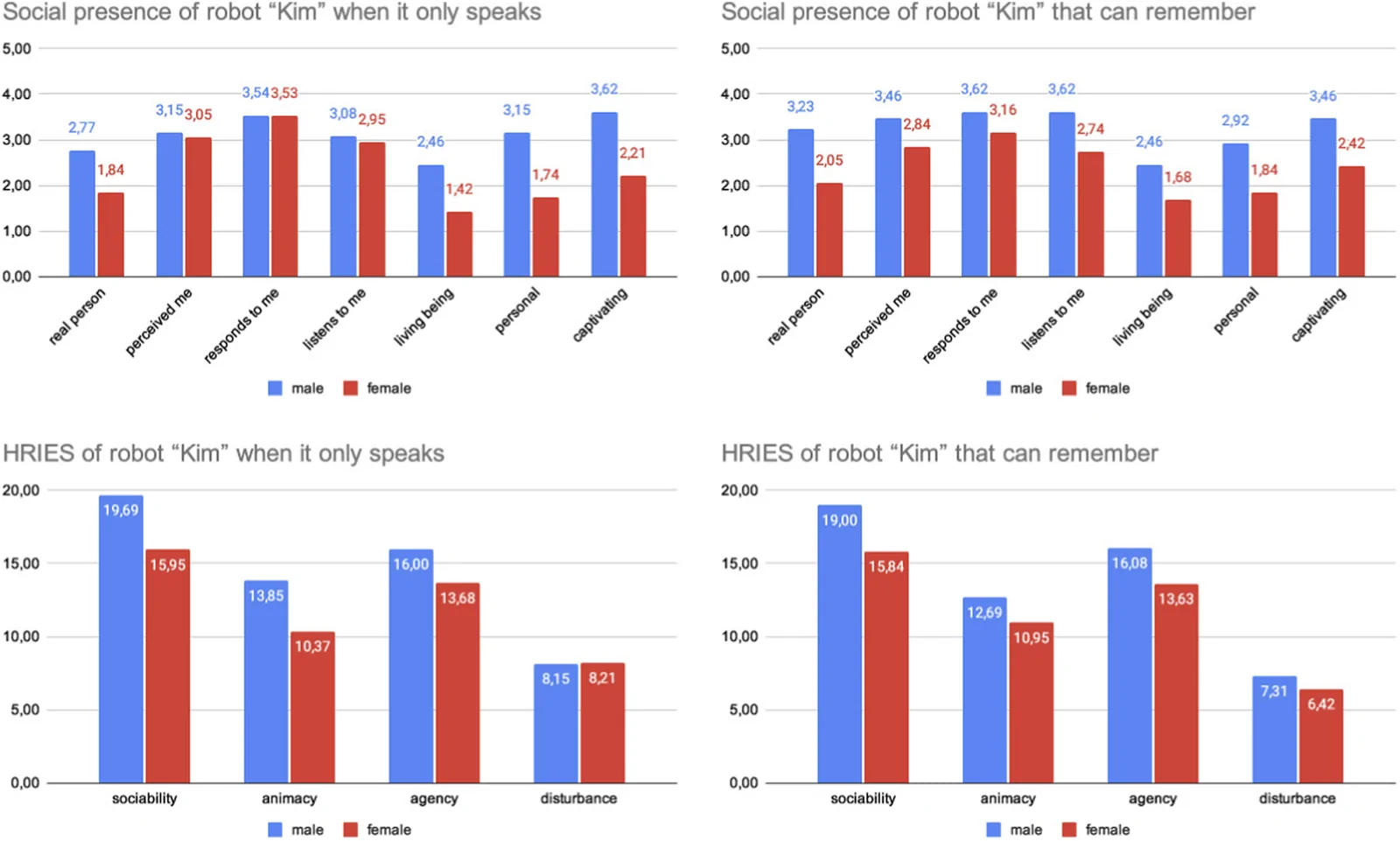
Evaluation results of the robot “Kim” by gender: Social presence and HRIES anthropomorphic dimensions.

## 5 Discussion

Sophisticated models of artificial intelligence, such as LLMs, have been shown to help create more realistic social interaction partners for people, particularly in the healthcare sector (Ragno et al., 2023). Social cognition influences the emotional and cognitive responses as well as the behavioral intentions of older adults, and socially supportive robots are capable of evoking a sense of social presence (Čaić et al., 2020). The sense of social presence influences how the robot is perceived in a caregiving role in terms of its humanlike-ness and intelligence (Kim et al., 2013). Furthermore, older users enjoy interacting with a robot more when they feel a stronger sense of social presence. Heerink et al. 2009, 2010b demonstrated that higher scores on expressiveness in conversation correlated with higher scores on social presence, and that social presence, in turn, correlated with the intention to use the system. This means that factors influencing social presence play an important role in the development of assistive agents for older adults.

Conversational expressiveness refers to the display of verbal and nonverbal interaction. Heerink et al. (2010b) found that users exhibited greater conversational expressiveness when interacting with a more sociable or expressive agent, which led to a stronger sense of social presence. Our assumption was that, in addition to the robot’s conversational expressiveness demonstrated by its conversational skills and accompanying gestures, further social cognitive abilities–such as visual perception of the interaction partner and memories of previous encounters and statements–would also lead to an increase in perceived social presence and perhaps also a higher level of anthropomorphism. This has tended to hold true, but only significantly so for one question regarding social presence, namely, whether the robot was paying attention to its human conversation partner, in case of the robot capable of visual perception. Several of our other questions regarding social presence also yielded higher scores in this case, though these were not statistically significant. Nevertheless, the tendency to perceive social presence as higher in a robot with visual capabilities suggests to us that visual perception is an important and relevant factor in determining how shared attention, shared mental models, and a common ground can be achieved in HRI. The fact that our results showed no significant change with regard to anthropomorphism suggests that the additional cognitive ability of vision had little effect on the perception of the humanoid robot Pepper’s naturalness and human-likeness.

The situation was different with regard to the cognitive ability of memory, which, in terms of social presence, increased the perception of the robot as a real person noticeably but not significantly. However, recalling details from a conversation that had taken place a few minutes earlier, during a second conversation following a pause, had a significant effect on the anthropomorphic dimension of disturbance. It is conceivable that the time interval was too short in this case to produce an impact on the social presence. Further studies involving repeated interactions over longer periods of time would be needed to determine this. Whether the reduced disturbance during the second conversation with Robot “Kim” can be explained solely by memory performance remains an open question. Other factors may also play a role here, such as familiarity with and experience of the robot based on the sequence of interactions. At least, no such significant change was observed in the interactions with Robot “Charlie”.

In line with existing research, our results show an increase in social presence when agents possess stronger sociable or expressive abilities. Specifically, however, it appears to depend on the exact nature of these enhanced abilities. For the two abilities we examined that contribute to more human-like behavior, there were at least clear differences in their effects on social presence and anthropomorphism. The study and comparison of other cognitive abilities related to social interactionss–such as emotion recognition and integration, as well as appropriate body language or tone of voice in robots–could shed additional light on how such abilities influence perceived social presence.

Even though the participants in our study did not belong to the age group of nursing home residents and the study was not conducted on-site at nursing facilities, we believe that our findings regarding a potential increase in social presence through the use of certain cognitive abilities are relevant to the important field of application for social assistive robots in the care and healthcare sectors, since a high level of social presence plays a significant role in this context. Future research could provide more clarity in this area. We had not expected any significant gender-specific differences in the perception of social presence and anthropomorphism, and therefore did not formulate any hypotheses on this topic. Although the differences can likely be explained by the varying professional backgrounds and interests of the majority of male and female participants, they nevertheless represent an interesting aspect that could also be examined in greater detail in future studies.

### 5.1 Limitations

This study was not conducted in a nursing care setting, and the study participants did not belong to the particularly vulnerable group of older adults in nursing homes, neither in terms of their specific living situation nor in terms of their age group. This raises the question of the extent to which the results of this study can be generalized to the intended target group. However, with younger participants and without the restrictive guidelines that would be required, for example, in nursing homes, this study represents a first step toward addressing the question of the impact of certain cognitive abilities in robots with regard to social presence and anthropomorphism.

Another limitation is the technical immaturity of the applications, particularly when it came to incorporating visual impressions into the conversation. Although the VLM typically provided accurate descriptions of the image content captured by the robot camera, the robot’s–from a human perspective–unnatural insistence on and repetition of visual details sometimes created an unfavorable impression of artificiality. Improved prompting for the LLM could address this issue. Occasionally, the VLM also produced hallucinations, meaning the robot mentioned things that were not actually there. The ability to recall specific details from the first conversation depended on correctly understanding the participant’s name in both the first and second conversation; furthermore, the clarity of these recollections was influenced by striking details mentioned in the first conversation, such that references to them stood out in the second conversation. Furthermore, the robots tended to ask many questions at once during a turn, which sometimes made it challenging for the participants to tailor their answers and focus on one or two aspects, since the robots were unable to fully process human responses that were too long due to turn-taking issues caused by pauses in human speech.

The range of evaluations provided by different study participants can likely be explained, at least in part, by their personal attitudes toward robots. When asked after completing their interactions with the robots, the vast majority of participants said that it had been an interesting experience for them. However, a few participants also expressed skepticism about the use of social robotics in caregiving. In addition, there was an imbalance between male and female study participants, which may have influenced the overall results. Some participants also criticized the robots’ monotonous, unnatural-sounding voice.

A major limitation in the assessment of gender-specific differences is probably due to the personal predispositions of both female and male participants, stemming from their different professional and academic backgrounds. Most female participants came from the fields of psychology, medicine, or nursing, whereas the majority of male participants had a more technical background. This fact and the resulting personal attitudes likely had a significant influence on how they evaluated their interactions with the robots. It is therefore possible that this is not, at least not solely, a gender-related difference, but rather one that can be explained by professional orientation, attitudes, and interests.

## 6 Conclusion

By examining the potential of cognitive abilities such as (i) unrestricted conversational skills, (ii) the incorporation of visual cues, and (iii) memories of individual encounters in robots during social interactions with humans, we were able to assess the social presence and anthropomorphic dimensions of such interactions. With only two exceptions, there were no statistically significant results. However, the findings showed that the ability to incorporate current visual impressions into a conversation tended to enhance the robot’s social presence from a human perspective. The study participants felt more acknowledged and perceived the robot’s remarks as specifically directed at them, which further reinforced the sense of a captivating conversation. The participants immediately noticed the robot’s additional visual capability, and most of them reacted with a mixture of surprise and amazement. That was certainly also because the robot explicitly made use of this capability.

In contrast, however, the robot’s ability to recall specific statements from a previous conversation did not lead to a meaningful increase in social presence–even though its memory was clearly recognized in some cases. Perhaps the short time interval between the conversations and the lack of long-term interaction played a role in why memory did not lead to substantial changes in perception. Sometimes the recalled details contained errors or were lost in the dialogue without being noticed. The effect of long-term memory on the perception of the robot’s social presence or anthropomorphism should be examined in more detail in a study involving repeated interactions over an extended period with the same participants. This may reveal whether the ability to remember over a longer period of time has an influence. The anthropomorphic dimensions measured using HRIES showed a significant change only in the case of memory, in terms of disturbance. We argue that the results of this study provide insights into the potential of various cognitive abilities to enhance the social presence of robots in HRI, which are likely to be particularly useful for the deployment of social robots as conversation partners in the care sector.

## Data Availability

The raw data supporting the conclusions of this article will be made available by the authors, without undue reservation.
